# (7-Chloro-2-oxo-2*H*-chromen-4-yl)methyl pyrrolidine-1-carbodi­thio­ate

**DOI:** 10.1107/S1600536813028080

**Published:** 2013-10-23

**Authors:** O. Kotresh, H. C. Devarajegowda, Arunkumar Shirahatti, K. Mahesh Kumar, N. M. Mahabhaleshwaraiah

**Affiliations:** a Department of Chemistry, Karnatak University’s Karnatak Science College, Dharwad, Karnataka 580 001, India; b Department of Physics, Yuvaraja’s College (Constituent College), University of Mysore, Mysore 570 005, Karnataka, India

## Abstract

In the title compound, C_15_H_14_ClNO_2_S_2_, the 2*H*-chromene ring system is essentially planar, with a maximum deviation of 0.0133 (10) Å. Three C atoms and their attached H atoms of the pyrrolidine ring are disordered [occupany ratio 0.874 (7):0.126 (7)] with both disorder components adopting a twisted conformation. The dihedral angle between the 2*H*-chromene ring system and the major occupancy component of the pyrrolidine ring is 89.45 (7)°. In the crystal, inversion dimers linked by pairs of C—H⋯S and C—H⋯O inter­actions generate *R* ^2^_2_(24) and *R* ^2^_2_(10) loops, respectively. Further C—H⋯O hydrogen bonds link the dimers into [100] chains. C—H⋯π inter­actions also occur and there is very weak π–π stacking [inter­planar spacing = 3.650 (5) Å; centroid–centroid distance = 4.095 (7) Å] between inversion-related chloro­benzene rings.

## Related literature

For biological applications of coumarins and di­thio­carbamates, see: Brillon (1992 ▶); Burns *et al.* (2010 ▶); Kawaii *et al.* (2001 ▶); Khan *et al.* (2004 ▶); Yu *et al.* (2003 ▶). For details of the synthesis and a related structure with comparison bond lengths, see: Mahabaleshwaraiah *et al.* (2012 ▶).
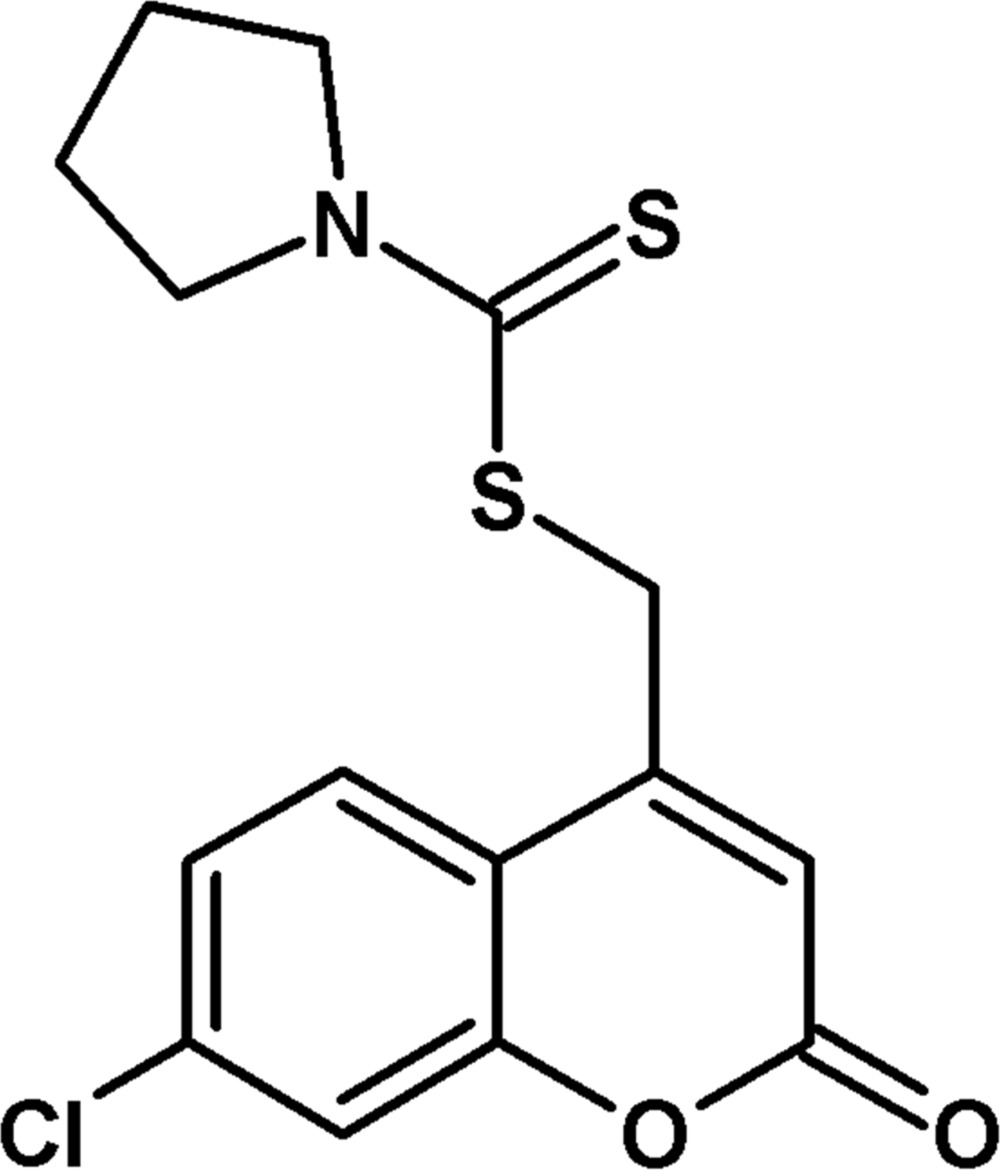


## Experimental

### 

#### Crystal data

C_15_H_14_ClNO_2_S_2_
*M* *_r_* = 339.84
Triclinic, 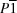

*a* = 7.9073 (2) Å
*b* = 9.2891 (2) Å
*c* = 10.8865 (2) Å
α = 84.474 (1)°
β = 79.798 (1)°
γ = 72.437 (1)°
*V* = 749.52 (3) Å^3^
*Z* = 2
Mo *K*α radiation
μ = 0.54 mm^−1^
*T* = 296 K
0.22 × 0.18 × 0.12 mm

#### Data collection

Bruker SMART CCD area-detector diffractometer
Absorption correction: multi-scan (*SADABS*; Sheldrick, 2007 ▶) *T*_min_ = 0.770, *T*_max_ = 1.000
16446 measured reflections
3417 independent reflections
3136 reflections with *I* > 2σ(*I*)
*R*_int_ = 0.023

#### Refinement

*R*[*F* ^2^ > 2σ(*F* ^2^)] = 0.031
*wR*(*F* ^2^) = 0.102
*S* = 1.18
3417 reflections
201 parameters
6 restraints
H-atom parameters constrained
Δρ_max_ = 0.28 e Å^−3^
Δρ_min_ = −0.31 e Å^−3^

### 

Data collection: *SMART* (Bruker, 2001 ▶); cell refinement: *SAINT* (Bruker, 2001 ▶); data reduction: *SAINT*; program(s) used to solve structure: *SHELXS97* (Sheldrick, 2008 ▶); program(s) used to refine structure: *SHELXL97* (Sheldrick, 2008 ▶); molecular graphics: *ORTEP-3 for Windows* (Farrugia, 2012 ▶); software used to prepare material for publication: *SHELXL97*.

## Supplementary Material

Crystal structure: contains datablock(s) I, global. DOI: 10.1107/S1600536813028080/pk2494sup1.cif

Structure factors: contains datablock(s) I. DOI: 10.1107/S1600536813028080/pk2494Isup2.hkl

Supplementary material file. DOI: 10.1107/S1600536813028080/pk2494Isup3.cml

966208

Additional supplementary materials:  crystallographic information; 3D view; checkCIF report

## Figures and Tables

**Table 1:** Hydrogen-bond geometry (Å, °) *Cg*4 is the centroid of the C7–C12 ring.

*D*—H⋯*A*	*D*—H	H⋯*A*	*D*⋯*A*	*D*—H⋯*A*
C9—H9⋯S3^i^	0.93	2.87	3.7910 (16)	170
C21—H21*B*⋯O5^ii^	0.97	2.60	3.3434 (19)	134
C16—H16*B*⋯*Cg*4^ii^	0.97	2.93	3.761 (1)	144

Symmetry codes: (i) 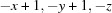; (ii) 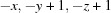.

**Fig. 1.**
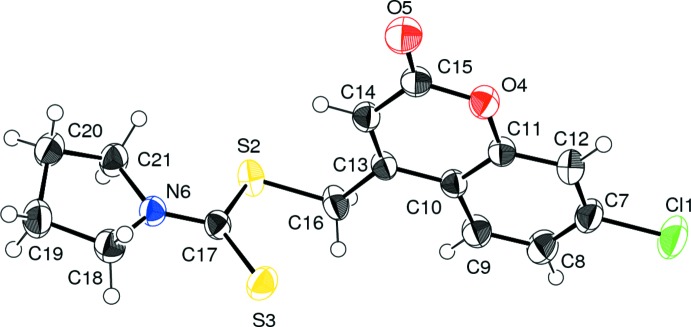
The molecular structure of the title compound. Displacement ellipsoids are drawn at the 50% probability level. Hydrogen atoms are shown as spheres of arbitrary radius. Only the major conformation of disorder at atom C20 is shown.

**Fig. 2.**
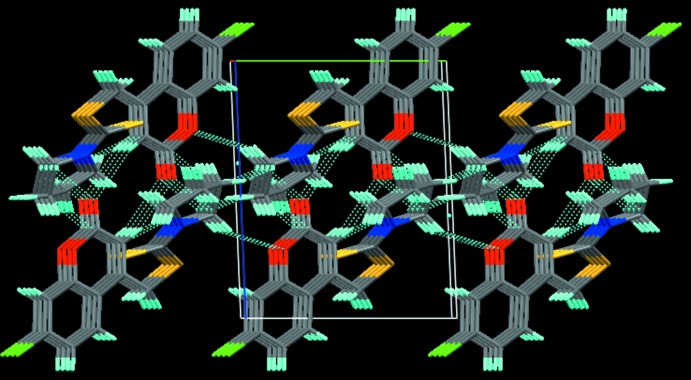
A packing diagram of the title compound.

## supplementary crystallographic information

### 1. Comment

Coumarin derivatives are an interesting class of heterocyclic systems, since the coumarin ring is an essential core moiety for a variety of natural and synthetic biologically active compounds. The biological activities include anticoagulation, antibiotic, antifungal, antipsoriasis, antitumor, anti-HIV, anti-inflammatory properties (Khan *et al.*, 2004; Kawaii *et al.*, 2001; Yu *et al.*, 2003). For a number of years, due to their important biological, biomedical and laser dye properties, coumarins have been the subject of a number of investigations, particularly on their photophysical characteristics, solvent effects on electronic absorption, structural properties and fluorescence spectra. The molecular manipulation of promising lead compounds is still a major line of approach to develop new drugs. It involves an effort to combine the separate pharmacophoric groups of similar activity into one compound, thereby affecting biological activity. The functionalization of the carbamate moiety is an effective technique for preparation of derivatives, which may have important therapeutic and biological properties (Brillon, 1992). In this regard, the introduction of new strategies to prepare dithiocarbamate derivatives with different substitution patterns at the thiol group has become a field of increasing interest in synthetic organic chemistry. There are several publications illustrating intramolecular or intermolecular oxygen sulfur exchange (Burns *et al.*, 2010).

The asymmetric unit of (7-chloro-2-oxo-2*H*-chromen-4-yl)methyl pyrrolidine-1-carbodithioate is shown in Fig. 1. The 2*H*- chromene (O4/C7–C15) ring system is planar, with a maximum deviation of 0.0133 (10) Å for atom C10, and the pyrrolidine ring adopts a twisted conformation. The dihedral angle between the 2*H*-chromene ring (O4/C7–C15) and the pyrrolidine ring (N6/C18–C21) is 89.45 (7)°. In the crystal, inversion dimers linked by pairs of C13—H13···S2 and C9—H9···O5 interactions generate *R*^2^_2_(24) and *R*^2^_2_(10) loops, respectively. Further C9—H9···O5 hydrogen bonds link the dimers into [100] chains. C16—H16B···π Cg4(C7–C12) (Table 1) interactions also occur and there is π-π Cg4 (C7–C12) stacking of inversion-related molecules, with interplanar spacings of 3.650 (5) Å and chlorobenzene ring centroid–centroid distances of 4.095 (7) Å. The packing of the molecules is depicted in Fig. 2. Disorder is observed at the pyrrolidine flap carbon (C20, C20') atom [occupancy ratio 0.876 (5):0.124 (5)].

### 2. Experimental

All the chemicals used were of analytical reagent grade and were used directly without further purification. The title compound was synthesized according to the reported method (Mahabaleshwaraiah *et al.*, 2012). The compound is recrystallized by ethanol-chloroform mixture. Colourless needles of the title compound were grown from a mixed solution of Ethanol/Chloroform (V/V = 2/1) by slow evaporation at room temperature. Yield = 74%, m.p. 445–447 K.

### Crystal data

**Table:**

C_15_H_14_ClNO_2_S_2_	*Z* = 2
*M**_r_* = 339.84	*F*(000) = 352
Triclinic, *P*1	*D*_x_ = 1.506 Mg m^−^^3^
Hall symbol: -P 1	Melting point: 447 K
*a* = 7.9073 (2) Å	Mo *K*α radiation, λ = 0.71073 Å
*b* = 9.2891 (2) Å	Cell parameters from 3417 reflections
*c* = 10.8865 (2) Å	θ = 1.9–27.5°
α = 84.474 (1)°	µ = 0.54 mm^−^^1^
β = 79.798 (1)°	*T* = 296 K
γ = 72.437 (1)°	Plate, colourless
*V* = 749.52 (3) Å^3^	0.22 × 0.18 × 0.12 mm

### Data collection

**Table:**

Bruker SMART CCD area-detector diffractometer	3417 independent reflections
Radiation source: fine-focus sealed tube	3136 reflections with *I* > 2σ(*I*)
Graphite monochromator	*R*_int_ = 0.023
ω and φ scans	θ_max_ = 27.5°, θ_min_ = 1.9°
Absorption correction: multi-scan (*SADABS*; Sheldrick, 2007)	*h* = −10→10
*T*_min_ = 0.770, *T*_max_ = 1.000	*k* = −12→12
16446 measured reflections	*l* = −14→14

### Refinement

**Table:**

Refinement on *F*^2^	Secondary atom site location: difference Fourier map
Least-squares matrix: full	Hydrogen site location: inferred from neighbouring sites
*R*[*F*^2^ > 2σ(*F*^2^)] = 0.031	H-atom parameters constrained
*wR*(*F*^2^) = 0.102	*w* = 1/[σ^2^(*F*_o_^2^) + (0.0617*P*)^2^ + 0.0857*P*] where *P* = (*F*_o_^2^ + 2*F*_c_^2^)/3
*S* = 1.18	(Δ/σ)_max_ = 0.004
3417 reflections	Δρ_max_ = 0.28 e Å^−^^3^
201 parameters	Δρ_min_ = −0.31 e Å^−^^3^
6 restraints	Extinction correction: *SHELXL97* (Sheldrick, 2008), Fc^*^=kFc[1+0.001xFc^2^λ^3^/sin(2θ)]^-1/4^
Primary atom site location: structure-invariant direct methods	Extinction coefficient: 0.049 (5)

### Special details

**Table:**

Experimental. IR (KBr, cm^-1^): 1731 (C=O), 1374 (C=S), 866(C—N). GCMS: m/e:339. ^1^H NMR (400 MHz, CDCl_3_, \?, p.p.m): 1.89 (m, 2H, CH_2_), 2.00 (m, 2H, CH_2_), 3.63(m, 2H, N—CH_2_), 3.77 (m, 2H, N—CH_2_), 4.81 (s,2*H*, C4—CH_2_),6.60 (s,1*H*, C3—H, Ar—H), 7.46 (m, H, Ar—H), 7.62 (m, 1H, Ar—H), 7.91 (s, 1H, Ar—H). Mol. Formula: C_15_H_14_Cl N O_2_S_2_. Elemental analysis: C, 53.01; H, 4.15; N, 4.12 (calculated); C, 52.98; H, 4.10; N, 4.09 (found).
Geometry. All s.u.'s (except the s.u. in the dihedral angle between two l.s. planes) are estimated using the full covariance matrix. The cell s.u.'s are taken into account individually in the estimation of s.u.'s in distances, angles and torsion angles; correlations between s.u.'s in cell parameters are only used when they are defined by crystal symmetry. An approximate (isotropic) treatment of cell s.u.'s is used for estimating s.u.'s involving l.s. planes.
Refinement. Refinement of *F*^2^ against ALL reflections. The weighted *R*-factor *wR* and goodness of fit *S* are based on *F*^2^, conventional *R*-factors *R* are based on *F*, with *F* set to zero for negative *F*^2^. The threshold expression of *F*^2^ > 2σ(*F*^2^) is used only for calculating *R*-factors(gt) *etc*. and is not relevant to the choice of reflections for refinement. *R*-factors based on *F*^2^ are statistically about twice as large as those based on *F*, and *R*- factors based on ALL data will be even larger.

### Fractional atomic coordinates and isotropic or equivalent isotropic displacement parameters (Å^2^)

**Table:**

	*x*	*y*	*z*	*U*_iso_*/*U*_eq_	Occ. (<1)
Cl1	−0.28700 (7)	1.07931 (5)	−0.14180 (5)	0.06885 (16)	
S2	0.38730 (4)	0.30580 (4)	0.21169 (3)	0.04235 (13)	
S3	0.54060 (5)	0.56231 (4)	0.24009 (4)	0.04455 (13)	
O4	−0.19899 (13)	0.79324 (11)	0.26803 (9)	0.0409 (2)	
O5	−0.17968 (15)	0.68451 (13)	0.45600 (9)	0.0526 (3)	
N6	0.61026 (14)	0.29726 (11)	0.36235 (10)	0.0338 (2)	
C7	−0.1734 (2)	0.92725 (17)	−0.05374 (14)	0.0459 (3)	
C8	−0.0243 (2)	0.8208 (2)	−0.11117 (14)	0.0520 (4)	
H8	0.0130	0.8294	−0.1968	0.062*	
C9	0.06851 (19)	0.70194 (18)	−0.04044 (13)	0.0461 (3)	
H9	0.1686	0.6303	−0.0791	0.055*	
C10	0.01464 (16)	0.68708 (14)	0.08890 (11)	0.0334 (3)	
C11	−0.13752 (16)	0.79680 (14)	0.14228 (12)	0.0345 (3)	
C12	−0.23300 (19)	0.91664 (16)	0.07278 (14)	0.0427 (3)	
H12	−0.3345	0.9880	0.1103	0.051*	
C13	0.10823 (15)	0.56837 (14)	0.17036 (11)	0.0324 (2)	
C14	0.04298 (16)	0.56563 (14)	0.29272 (12)	0.0354 (3)	
H14	0.1019	0.4881	0.3444	0.043*	
C15	−0.11598 (17)	0.67905 (15)	0.34721 (12)	0.0370 (3)	
C16	0.27739 (18)	0.45665 (17)	0.11144 (13)	0.0425 (3)	
H16A	0.3621	0.5117	0.0751	0.051*	
H16B	0.2488	0.4124	0.0435	0.051*	
C17	0.52248 (15)	0.38901 (13)	0.27966 (11)	0.0327 (2)	
C18	0.73425 (19)	0.33810 (16)	0.42944 (14)	0.0418 (3)	
H18A	0.6702	0.4194	0.4850	0.050*	
H18B	0.8263	0.3692	0.3714	0.050*	
C19	0.8152 (2)	0.1946 (2)	0.50227 (18)	0.0558 (4)	0.874 (7)
H19A	0.8384	0.2173	0.5817	0.067*	0.874 (7)
H19B	0.9270	0.1359	0.4555	0.067*	0.874 (7)
C20	0.6762 (3)	0.1094 (2)	0.5219 (2)	0.0480 (6)	0.874 (7)
H20A	0.7317	0.0016	0.5335	0.058*	0.874 (7)
H20B	0.5860	0.1442	0.5941	0.058*	0.874 (7)
C21	0.59481 (19)	0.14500 (14)	0.40330 (14)	0.0413 (3)	0.874 (7)
H21A	0.6606	0.0722	0.3409	0.050*	0.874 (7)
H21B	0.4702	0.1451	0.4192	0.050*	0.874 (7)
C19'	0.8152 (2)	0.1946 (2)	0.50227 (18)	0.0558 (4)	0.126 (7)
H19C	0.9449	0.1723	0.4892	0.067*	0.126 (7)
H19D	0.7715	0.2069	0.5908	0.067*	0.126 (7)
C20'	0.765 (2)	0.0709 (13)	0.4603 (17)	0.052 (4)	0.126 (7)
H20C	0.7430	0.0024	0.5301	0.062*	0.126 (7)
H20D	0.8604	0.0147	0.3986	0.062*	0.126 (7)
C21'	0.59481 (19)	0.14500 (14)	0.40330 (14)	0.0413 (3)	0.126 (7)
H21C	0.5907	0.0899	0.3331	0.050*	0.126 (7)
H21D	0.4883	0.1506	0.4648	0.050*	0.126 (7)

### Atomic displacement parameters (Å^2^)

**Table:**

	*U*^11^	*U*^22^	*U*^33^	*U*^12^	*U*^13^	*U*^23^
Cl1	0.0754 (3)	0.0589 (3)	0.0702 (3)	−0.0126 (2)	−0.0342 (2)	0.0265 (2)
S2	0.0401 (2)	0.0358 (2)	0.0502 (2)	−0.00229 (14)	−0.01778 (15)	−0.00600 (14)
S3	0.0498 (2)	0.03124 (19)	0.0489 (2)	−0.00884 (14)	−0.00686 (15)	0.00535 (14)
O4	0.0416 (5)	0.0369 (5)	0.0365 (5)	−0.0022 (4)	−0.0011 (4)	−0.0042 (4)
O5	0.0544 (6)	0.0595 (7)	0.0336 (5)	−0.0059 (5)	0.0024 (4)	−0.0043 (5)
N6	0.0334 (5)	0.0290 (5)	0.0396 (5)	−0.0082 (4)	−0.0089 (4)	−0.0002 (4)
C7	0.0503 (8)	0.0430 (7)	0.0463 (8)	−0.0135 (6)	−0.0197 (6)	0.0118 (6)
C8	0.0541 (8)	0.0620 (10)	0.0353 (7)	−0.0124 (7)	−0.0084 (6)	0.0084 (6)
C9	0.0411 (7)	0.0555 (8)	0.0344 (7)	−0.0050 (6)	−0.0044 (5)	0.0005 (6)
C10	0.0314 (6)	0.0366 (6)	0.0319 (6)	−0.0091 (5)	−0.0064 (4)	−0.0009 (5)
C11	0.0342 (6)	0.0342 (6)	0.0348 (6)	−0.0092 (5)	−0.0062 (5)	−0.0012 (5)
C12	0.0413 (7)	0.0355 (6)	0.0483 (8)	−0.0050 (5)	−0.0109 (6)	0.0000 (5)
C13	0.0281 (5)	0.0350 (6)	0.0337 (6)	−0.0069 (4)	−0.0074 (4)	−0.0023 (5)
C14	0.0336 (6)	0.0368 (6)	0.0343 (6)	−0.0070 (5)	−0.0074 (5)	−0.0001 (5)
C15	0.0373 (6)	0.0393 (6)	0.0329 (6)	−0.0100 (5)	−0.0036 (5)	−0.0027 (5)
C16	0.0351 (6)	0.0494 (8)	0.0343 (6)	0.0026 (5)	−0.0071 (5)	−0.0051 (5)
C17	0.0295 (5)	0.0298 (6)	0.0342 (6)	−0.0028 (4)	−0.0015 (4)	−0.0042 (4)
C18	0.0424 (7)	0.0397 (7)	0.0484 (7)	−0.0152 (5)	−0.0158 (6)	0.0004 (6)
C19	0.0535 (9)	0.0533 (9)	0.0678 (10)	−0.0193 (7)	−0.0311 (8)	0.0153 (8)
C20	0.0510 (12)	0.0429 (10)	0.0519 (12)	−0.0158 (9)	−0.0173 (10)	0.0130 (8)
C21	0.0450 (7)	0.0286 (6)	0.0521 (8)	−0.0107 (5)	−0.0153 (6)	0.0037 (5)
C19'	0.0535 (9)	0.0533 (9)	0.0678 (10)	−0.0193 (7)	−0.0311 (8)	0.0153 (8)
C20'	0.045 (8)	0.042 (6)	0.062 (10)	−0.002 (5)	−0.018 (7)	0.011 (6)
C21'	0.0450 (7)	0.0286 (6)	0.0521 (8)	−0.0107 (5)	−0.0153 (6)	0.0037 (5)

### Geometric parameters (Å, º)

**Table:**

Cl1—C7	1.7320 (14)	C13—C14	1.3413 (17)
S2—C17	1.7822 (13)	C13—C16	1.5047 (17)
S2—C16	1.7934 (14)	C14—C15	1.4487 (17)
S3—C17	1.6678 (12)	C14—H14	0.9300
O4—C11	1.3699 (15)	C16—H16A	0.9700
O4—C15	1.3796 (16)	C16—H16B	0.9700
O5—C15	1.2015 (16)	C18—C19	1.509 (2)
N6—C17	1.3172 (16)	C18—H18A	0.9700
N6—C18	1.4731 (16)	C18—H18B	0.9700
N6—C21	1.4757 (16)	C19—C20	1.513 (2)
C7—C12	1.379 (2)	C19—H19A	0.9700
C7—C8	1.383 (2)	C19—H19B	0.9700
C8—C9	1.377 (2)	C20—C21	1.508 (2)
C8—H8	0.9300	C20—H20A	0.9700
C9—C10	1.4031 (18)	C20—H20B	0.9700
C9—H9	0.9300	C21—H21A	0.9700
C10—C11	1.3966 (17)	C21—H21B	0.9700
C10—C13	1.4498 (16)	C20'—H20C	0.9700
C11—C12	1.3837 (18)	C20'—H20D	0.9700
C12—H12	0.9300		
			
C17—S2—C16	102.76 (7)	C13—C16—H16A	108.1
C11—O4—C15	121.50 (10)	S2—C16—H16A	108.1
C17—N6—C18	123.15 (11)	C13—C16—H16B	108.1
C17—N6—C21	125.66 (11)	S2—C16—H16B	108.1
C18—N6—C21	111.16 (10)	H16A—C16—H16B	107.3
C12—C7—C8	121.59 (13)	N6—C17—S3	123.58 (10)
C12—C7—Cl1	118.87 (12)	N6—C17—S2	112.65 (9)
C8—C7—Cl1	119.53 (12)	S3—C17—S2	123.75 (7)
C9—C8—C7	119.44 (13)	N6—C18—C19	103.94 (11)
C9—C8—H8	120.3	N6—C18—H18A	111.0
C7—C8—H8	120.3	C19—C18—H18A	111.0
C8—C9—C10	121.17 (14)	N6—C18—H18B	111.0
C8—C9—H9	119.4	C19—C18—H18B	111.0
C10—C9—H9	119.4	H18A—C18—H18B	109.0
C11—C10—C9	117.20 (12)	C18—C19—C20	105.04 (12)
C11—C10—C13	118.25 (11)	C18—C19—H19A	110.7
C9—C10—C13	124.53 (12)	C20—C19—H19A	110.7
O4—C11—C12	115.98 (11)	C18—C19—H19B	110.7
O4—C11—C10	121.51 (11)	C20—C19—H19B	110.7
C12—C11—C10	122.50 (12)	H19A—C19—H19B	108.8
C7—C12—C11	118.08 (13)	C21—C20—C19	103.84 (14)
C7—C12—H12	121.0	C21—C20—H20A	111.0
C11—C12—H12	121.0	C19—C20—H20A	111.0
C14—C13—C10	119.02 (11)	C21—C20—H20B	111.0
C14—C13—C16	123.85 (11)	C19—C20—H20B	111.0
C10—C13—C16	117.11 (11)	H20A—C20—H20B	109.0
C13—C14—C15	122.38 (12)	N6—C21—C20	103.91 (11)
C13—C14—H14	118.8	N6—C21—H21A	111.0
C15—C14—H14	118.8	C20—C21—H21A	111.0
O5—C15—O4	116.97 (12)	N6—C21—H21B	111.0
O5—C15—C14	125.72 (13)	C20—C21—H21B	111.0
O4—C15—C14	117.28 (11)	H21A—C21—H21B	109.0
C13—C16—S2	116.78 (9)	H20C—C20'—H20D	108.7
			
C12—C7—C8—C9	−0.7 (2)	C11—O4—C15—O5	−179.57 (12)
Cl1—C7—C8—C9	178.41 (13)	C11—O4—C15—C14	2.14 (17)
C7—C8—C9—C10	−0.1 (3)	C13—C14—C15—O5	−178.77 (14)
C8—C9—C10—C11	0.6 (2)	C13—C14—C15—O4	−0.64 (19)
C8—C9—C10—C13	−177.89 (13)	C14—C13—C16—S2	3.91 (18)
C15—O4—C11—C12	179.60 (12)	C10—C13—C16—S2	−177.56 (9)
C15—O4—C11—C10	−1.62 (18)	C17—S2—C16—C13	−84.97 (11)
C9—C10—C11—O4	−179.01 (12)	C18—N6—C17—S3	0.16 (17)
C13—C10—C11—O4	−0.43 (18)	C21—N6—C17—S3	178.05 (10)
C9—C10—C11—C12	−0.31 (19)	C18—N6—C17—S2	178.53 (10)
C13—C10—C11—C12	178.27 (12)	C21—N6—C17—S2	−3.57 (16)
C8—C7—C12—C11	1.0 (2)	C16—S2—C17—N6	177.14 (9)
Cl1—C7—C12—C11	−178.15 (10)	C16—S2—C17—S3	−4.49 (9)
O4—C11—C12—C7	178.31 (12)	C17—N6—C18—C19	−174.43 (12)
C10—C11—C12—C7	−0.5 (2)	C21—N6—C18—C19	7.40 (16)
C11—C10—C13—C14	1.87 (18)	N6—C18—C19—C20	−26.24 (19)
C9—C10—C13—C14	−179.66 (13)	C18—C19—C20—C21	35.3 (2)
C11—C10—C13—C16	−176.73 (11)	C17—N6—C21—C20	−163.79 (15)
C9—C10—C13—C16	1.74 (19)	C18—N6—C21—C20	14.32 (17)
C10—C13—C14—C15	−1.34 (18)	C19—C20—C21—N6	−30.1 (2)
C16—C13—C14—C15	177.16 (12)		

### Hydrogen-bond geometry (Å, º)

Cg4 is the centroid of the C7–C12 ring.

**Table:**

*D*—H···*A*	*D*—H	H···*A*	*D*···*A*	*D*—H···*A*
C9—H9···S3^i^	0.93	2.87	3.7910 (16)	170
C21—H21*B*···O5^ii^	0.97	2.60	3.3434 (19)	134
C16—H16*B*···*Cg*4^ii^	0.97	2.93	3.761 (1)	144

Symmetry codes: (i) −*x*+1, −*y*+1, −*z*; (ii) −*x*, −*y*+1, −*z*+1.
